# Theoretical Investigations of the Photophysical Properties of Star-Shaped π-Conjugated Molecules with Triarylboron Unit for Organic Light-Emitting Diodes Applications

**DOI:** 10.3390/ijms18102178

**Published:** 2017-10-18

**Authors:** Ruifa Jin, Xiaofei Zhang, Wenmin Xiao, Dongmei Luo

**Affiliations:** 1 College of Chemistry and Chemical Engineering, Chifeng University, Chifeng 024000, China; 15849630086@126.com (X.Z.); xiaowenmin6868@163.com (W.X.); luodongmei1976@163.com (D.L.); 2 Inner Mongolia Key Laboratory of Photoelectric Functional Materials, Chifeng University, Chifeng 024000, China

**Keywords:** organoborons molecules, electronic and optical properties, charge transport property, organic light-emitting diodes (OLEDs)

## Abstract

The density functional theory (DFT) and time-dependent DFT (TD-DFT) methodologies have been applied to explore on a series of star-shaped π-conjugated organoboron systems for organic light-emitting diode (OLED) materials. The compounds under investigation consist of benzene as π-bridge and different core units and triarylboron end groups. Their geometry structures, frontier molecular orbital (FMO) energies, absorption and fluorescence spectra, and charge transport properties have been investigated systematically. It turned out that the FMO energy levels, the band gaps, and reorganization energies optical are affected by the introduction of different core units and triarylboron end groups. The results suggest that the designed compounds are expected to be promising candidates for luminescent materials. Furthermore, they can also serve as hole and/or electron transport materials for OLEDs.

## 1. Introduction

Organic π-conjugated luminophors have recently stirred great attention because of their potential applications as molecular probes and switches, organic light-emitting diodes (OLEDs), and solid-state light sources [1,2,3,4,5,6,7]. Particularly, π-conjugated organoboron systems have attracted considerable interest for OLEDs due to their outstanding properties, such as their high photo and chemical stability, good charge transport property, and high fluorescence quantum yields [8,9,10]. Unfortunately, the efficiency of OLEDs is still comparatively low, a shortcoming that restricts their commercialization application. To promote the efficiency of OLEDs, the luminescence and charge transport properties have become the two top-priority issues for OLEDs materials [11]. It has therefore become an urgent need to find novel materials with high luminous efficiency and carrier mobility for use in OLEDs [12,13]. Since the vacant p_π_ orbital of boron and the extended π-system, the π-conjugated three-coordinate organoborons exhibited strong electron accepting ability [14] and good charge transport properties [15]. In addition, variations in the chromophore part of the molecule would influence the frontier molecular orbital (FMO) energy levels and thereby the color of emission [16,17]. Namely, the FMO energy levels and the color of emission can be modulated by the introduction of different groups in the molecules. Particularly, the introduction of an electron-donating group can improve the intense luminescence and carrier mobility. Therefore, π-conjugated organoboron systems are expected to be the promising candidates for bifunctional OLEDs materials with strong fluorescence and high carrier mobility [18,19]. Moreover, comparing with a linear one, π-conjugated materials with star-shaped architecture possess the prime virtues of their good charge transport property. This kind material can enhance the π–π stacking of conjugated arms because of their high structural symmetry and planarity. Furthermore, they can also cause no an ordered, long-range, and coplanar π–π stacking due to their steric hindrance [20,21]. On the other hand, theoretical investigation also plays an essential role in the design and synthesis of materials. A number of studies demonstrate the interplay between theory and experiment, which is capable of providing useful insights into the understanding of the nature of molecules [21,22,23]. Recently, it has been reported that triarylboron-systems π-conjugated three-coordinate organoborons possessed excellent optical and charge transfer properties for use in OLEDs [14].

**Scheme 1:**
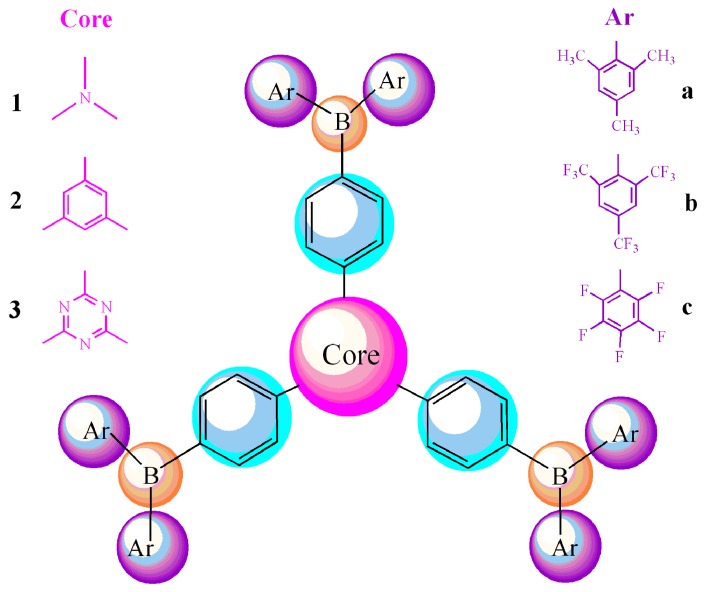
Molecular structures of the investigated molecules.

In this contribution, with the aim to enhance the electronic, optical, and charge transfer properties of materials, we have designed new π-conjugated three-coordinate organoborons with benzene π-bridge fragments (BBs), different core fragments (CFs), and triarylboron end groups (TBGs) (Scheme 1). The purpose of this kind molecular structure is to investigate the relationship between topologic structure and electronic, optical, and charge transfer properties, providing a demonstration for the rational design of a novel candidate for luminescent and charge transport materials for OLEDs. By applying density functional theory (DFT) and time-dependent DFT (TD-DFT) methodology, we calculated the FMO (HOMO and LUMO) energies (*E*_HOMO_ and *E*_LUMO_), the HOMO–LUMO gaps (*E*_g_), the absorption and fluorescence spectra, and the reorganization energies of designed molecules.

## 2. Results and Discussion

### 2.1. Frontier Molecular Orbitals

**Figure 1:**
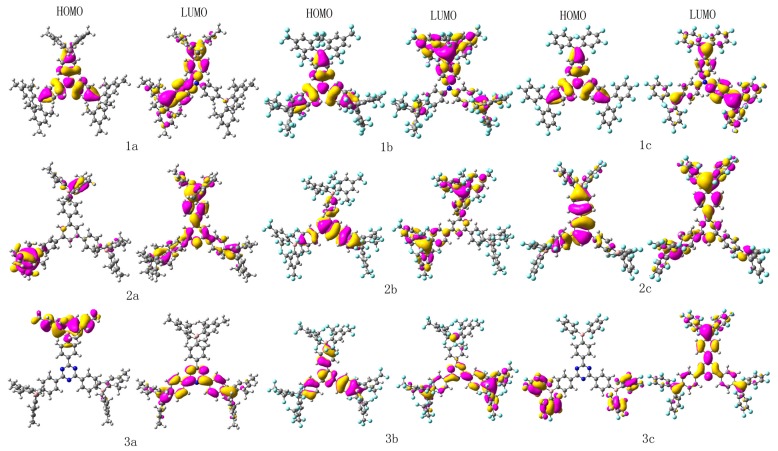
The electronic density contours of the frontier orbitals for the studied compounds at the PBE0/6-31G(d,p) level.

With the aim to investigate the optical and electronic properties, the FMOs of the designed molecules were examined. The distributions HOMOs and LUMOs of the designed molecules are shown in Figure 1. The *E*_HOMO_ and *E*_LUMO_, *E*_g_, and the contributions of individual fragments (in %) to the FMOs of the designed molecules are given in Table 1. From Figure 1, one can see that both HOMOs and LUMOs exhibit π characteristics. For **1a**–**c**, **2b**, **2c**, and **3b**, both the HOMOs and LUMOs are spread over the CFs, BBs, and TBGs of the molecules. However, the HOMOs are mainly localized on TBGs, while their LUMOs are spread over the whole molecule for **2a**, **3a**, and **3c**. Inspection of Table 1 reveals clearly that the HOMOs of **1a**–**c**, **2b**, **2c**, and **3b** are mainly localized on CFs and BBs, with minor contributions from the TBGs. Their LUMOs are mainly centralized on BBs and TBGs, with minimal density on CFs. The sum contributions of CFs and BBs for HOMOs are larger than 87.6%, while the contributions of TBGs are within 15.7%. For **2a**, **3a**, and **3c**, the contributions of TBGs for HOMOs are larger than 90.7%, while the sum contributions of CFs and BBs are within 9.3%. On the other hand, the sum contributions of BBs and TBGs of LUMOs are larger than 84.3%, while the contributions of CFs are within 12.4%. These results reveal that different configurations of the CF and TBG building blocks have obvious effects on the distribution of FMOs. Furthermore, the contributions of CFs and BBs for **1a**, **1c**, **2b**, **2**c, and **3b** are decreased, while the corresponding contributions of TBGs are increased compared with those of HOMOs, respectively. The percentages of charge transfer from CFs and BBs to TBGs are 43.2, 53.9, 73.1, 53.0, and 55.2%, respectively. In contrast, the contributions of CFs and BBs for **2a**, **3a**, and **3c** are increased, while the corresponding contributions of TBGs are decreased compared with those of HOMOs. The corresponding charge transfer from TBGs to CFs and BBs are 38.3, 65.5, and 52.7%, respectively. For **1b**, the contribution of CFs is decreased, and the contributions of BBs and TBGs are increased compared with those of HOMOs, respectively. These results reveal that the excitation of the electron from the HOMOs to the LUMOs causes the electronic density to flow mainly from CFs and BBs to TBGs for **1a**, **1c**, **2b**, **2**c, and **3b**. For **2a**, **3a**, and **3c**, the electronic densities mainly flow from TBG fragments to CFs and BBs. The electronic densities mainly flow from CFs to BBs and TBGs for **1b**. It suggests that CFs and BBs serve as donors and TBGs serve as acceptors for **1a**, **1c**, **2b**, **2**c, and **3b**. The CFs serve as the donor, and BBs and TBGs serve as acceptors for **1b**. In general, TBGs serve as electron acceptors. Interestingly, for **2a**, **3a**, and **3c**, the TBGs serve as donors, and CFs and BBs serve as acceptors. It is well known that the photophysical properties of intramolecular charge transfer are highly dependent on the electron donor/acceptor strength [24,25]. For **2a** and **3a**, the reasons may be due to the electron donating of two mesitylene groups in TBGs, which strengthens the electron donating abilities of (Mes)_2_B compared with those of CFs and BBs, respectively. For **3c**, the TBGs serve as donors because of the strongly mesomeric effect of fluorine atoms of two pentafluorobenzenes groups in TBGs, which increases the electron donating ability of (PFB)_2_B compared with those of CFs and BBs.

It is commonly known that the *E*_HOMO_, *E*_LUMO_, and *E*_g_ are closely related to the optical and electronic properties of molecules. For the designed molecules with nitrogen as core, **1a**–**c**, the *E*_HOMO_ and *E*_LUMO_ are in the order **1a** > **1b** > **1c** and **1a** > **1c** > **1b**, respectively. Therefore, the *E*_g_ values is in the sequence **1a** > **1c** > **1b**. This shows that Molecule **1a** with ((mesitylene)_2_B, (Mes)_2_B) TBGs possesses higher *E*_HOMO_ and *E*_LUMO_ and larger *E*_g_ in comparison to molecules with ((1,3,5-tris(trifluoromethyl)benzene)_2_B, (FMes)_2_B), or ((1,2,3,4,5-pentafluorobenzene)_2_B, (PFB)_2_B) as TBGs. The reason is that –CF_3_ and –F in TBGs (FMes)_2_B and (PFB)_2_B is electron-withdrawing in nature, while –CH_3_ in TBG (Mes)_2_B exhibit an electron-donating character. The electron-withdrawing groups –CF_3_ and –F are enable the electronic density to flow from donors to acceptors, while the electron-donating group –CH_3_ in TBG (Mes)_2_B is unfavorable to the charge transfer. Moreover, the mesomeric effect of –F in (PFB)_2_B strengthens the electron donating ability of (PFB)_2_B in **1c**. Therefore, the electron-withdrawing strength of (PFB)_2_B is weaker than that of (FMes)_2_B in **1b**. As a consequence, the electronic density to flow from donors to acceptors for **1b** is more fluent than that of **1c**. Thus, the E_HOMO_ of 1b is higher than that of **1c**, whereas the E_LUMO_ of **1b** is lower than that of **1c**. Similar phenomena are found for the designed molecules with benzene as core, **2a**–**c**. For the designed molecules with 1,3,5-triazine as core, **3a**–**c**, the sequences of *E*_HOMO_ and *E*_LUMO_ are **3a** > **3c** > **3b** and **3a** > **3b** >**3c**, respectively. As a result, the order of *E*_g_ is **3b** > **3c** > **3a**. This shows that Molecule **3a** with (Mes)_2_B end group possesses higher *E*_LUMO_ and *E*_HOMO_ values and thus a smaller *E*_g_ in comparison with (FMes)_2_B and (PFB)_2_B as the end group. These can be explained by the fact that the electron-donating group –CH_3_ in the (Mes)_2_B end group of **3a** improves charge transfer, while the electron-withdrawing group 1,3,5-triazine core fragment of **3b** may prevent the electronic density from flowing from donor to acceptor fluently. These results indicate that the electron-withdrawing or electron-donating characters of CFs and TBGs significantly influence the *E*_g_ values for the designed molecules.

### 2.2. Absorption and Fluorescence Spectra

The longest absorption wavelengths *λ*_abs_, electronic transitions, main assignments, and the oscillator strength *f* for the designed molecules are listed in Table 2. The absorptions of **1a**–**c**, **2a**–**c**, and **3b** are assigned to the S_0_ → S_1_ electronic transitions, while the absorption of **3a** and **3c** are assigned to the S_0_ → S_2_ and S_0_ → S_3_ electronic transitions, respectively. The longest absorption of **1a**–**c**, **2c**, and **3b** essentially mainly originates from HOMO → LUMO transition, while the corresponding absorption of **2b** and **3c** are mainly attributed to HOMO → LUMO+1 transitions. However, the transitions of absorption for **2a** and **3a** correspond to HOMO−2 → LUMO+1 transitions. Moreover, the sequence of *λ*_abs_ is **1b** > **1c** > **1a** > **3a** > **2b** > **2a** > **2c** > **3c** > **3b**. It indicates that the designed molecules with nitrogen as core show longer absorption wavelengths than those with benzene and 1,3,5-triazine as cores. Furthermore, **2c** has the largest oscillator strengths in the designed molecules. **1a**–**c**, **2b**, and **3b** have large oscillator strengths, while the oscillator strengths of **2a**, **3a**, and **3c** are smaller than those of other molecules. This indicates that **1a**–**c**, **2b**, **2c**, and **3b** may exhibit larger absorption intensity than those of **2a**, **3a**, and **3c**.

The longest fluorescence wavelengths *λ*_fl_, electronic transitions, main assignments, and the oscillator strength *f* of the designed molecules are given in Table 3. The fluorescence of **1a**, **2b**, **3a**, and **3b** are assigned to the S_1_ → S_0_ electronic transitions, while the fluorescence of **1b**, **1c**, **2a**, and **2c** are assigned to the S_2_ → S_0_ electronic transitions. For **3c**, the fluorescence is assigned to the S_3_ → S_0_ electronic transition. The LUMO → HOMO transitions play a dominant role for the fluorescence of **1a**, **2a**, **2b**, **3a**, and **3b**. However, the fluorescence’s of **1b**, **1c**, and **2c** are mainly attributed to LUMO+1 → HOMO transitions. For **3c**, the fluorescence is mainly attributed to a LUMO → HOMO−5 transition. Furthermore, **1a**–**c**, **2a**, and **2c** have larger oscillator strengths than those of **3a**–**c**. The oscillator strength value of **2b** is equal to that of **2b**. This implies that **1a**–**c**, **2a**, and **2c** exhibit fluorescence spectra that is stronger than those of **3a**–**c**. This suggests that the designed molecules have greater fluorescence intensity and are promising luminescent materials for OLEDs, particularly for **1a**–**c**, **2a**, and **2c**.

### 2.3. Reorganization Energy

It is noteworthy that the small reorganization energy could be beneficial for a significant charge transport [26,27]. The reorganization energies for hole and electron of the designed molecules are calculated and listed in Table 4. Usually, *N*,*N*′-diphenyl-*N*,*N*′-bis(3-methlphenyl)-(1,1′-biphenyl)-4,4′-diamine (TPD) (*λ*_h_ = 0.290 eV) [28] and tris(8-hydroxyquinolinato)aluminum(III) (Alq3) (*λ*_e_ = 0.276 eV) [29] are the typical hole and electron transport materials, respectively. From Table 4, one can find that the *λ*_h_ values of **1a**, **1c**, **2a**, **2c**, and **3a**–**c** are smaller than that of TPD. However, the *λ*_h_ values of **1b** and **2b** are larger than that of TPD. This indicates that the hole transfer rates of **1a**, **1c**, **2a**, **2c**, and **3a**–**c** may be higher, while the corresponding hole transfer rates of **1b** and **2b** might be lower than that of TPD. On the other hand, the calculated *λ*_e_ values of the designed molecules (0.082–0.263 eV) are smaller than that of Alq3. This suggests that the electron transfer rates of the designed molecules might be higher than that of Alq3. The *λ*_h_ values are predicted in the order **1b** > **1a** > **1c** for **1a**–**c**. The sequences of *λ*_h_ values are **2b** > **2c** > **2a** and **3b** > **3c** > **3a** for **2a**–**c** and **3a**–**c**, respectively. This suggests that the (Mes)_2_B and (PFB)_2_B end groups can increase the hole transfer rates, while the (FMes)_2_B end groups decrease the hole transfer rates for the designed molecules. For the *λ*_e_, the order of **1a**–**c** and **2a**–**c** are **1a** > **1b** > **1c** and **2a** > **2b** > **2c**, respectively, while the sequences of **3a**–**c** is **3a** > **3c** > **3b**. This implies that molecules with (FMes)_2_B and (PFB)_2_B end groups have greater electron transfer rates than those with (Mes)_2_B end groups, respectively. Therefore, **1a**, **1c**, **2a**, **2c**, and **3a**–**c** are expected to be promising candidates for hole as well as electron transport materials, whereas **1b** and **2b** can serve as electron transport materials only.

## 3. Materials and Methods

### Computational Methods

All calculations were performed using Gaussian 09 code [30]. Geometry optimizations in the ground state (S_0_) and the lowest singlet excited state (S_1_) were carried out using the PBE0 and TD-PBE0 functionals, respectively, together with the 6-31G(d,p) basis set. On the basis of the optimized S_0_ and S_1_ geometries, the absorption and fluorescence spectra were predicted with the TD-PBE0/6-31G(d,p) method. With the aim to attest to the validity of the selected approach, *N*-(4-(dimesitylboryl)phenyl)-*N*-phenylbenzenamine (DBPB) was taken as an example because its geometric structure is similar to those of the designed molecules. The molecular structure of DBPB is presented in Figure S1. The geometry optimizations of DBPB in S_0_ and S_1_ states were carried out via the DFT and TD-DFT method using the 6-31G(d,p) basis set, respectively. The absorptions and fluorescence spectra were predicted using TD-DFT with the 6-31G(d,p) basis set based on the optimized geometries in S_0_ and S_1_ states, respectively. The various functionals for all DFT and TD-DFT computations include B3LYP, PBE0, CAM-B3LYP, wB97XD, and M062X. The longest absorptions and fluorescence wavelengths of DBPB are listed in Table S1. The results displayed in Table S1 show that the TD-PBE0/6-31G(d,p) method provided a better agreement with the reported experimental observations [14] than those obtained with other methods. Furthermore, our previous work [31] and other reports in the literature [32] suggest that PBE0 appeared notably adapted to organoboron compounds. Therefore, geometry optimizations, band gaps *E*_g_, and the absorption and fluorescence properties of the designed molecules were carried out with the PBE0/6-31G(d,p) method.

According to Marcus electron transfer theory [26,27], two key factors that determine the charge transfer rates are the charge transfer coupling integral *V* and the reorganization energy *λ*. The *V* values can be obtained through the crystal data. However, the crystal structures of the designed molecules are unavailable and may be non-crystal. Therefore, we investigate the charge transport property of the designed molecules using reorganization energies *λ*. The *λ* can be partitioned into internal and external contributions. The internal reorganization energy *λ*_int_ is induced by structural change between ionic and neutral states [33]. The external reorganization energy *λ*_ext_ arises from the surrounding media in bulk materials. Generally, the predicted value of *λ*_ext_ in pure organic phases is not only small but also much smaller than its *λ*_int_ [34,35,36]. Therefore, we focus on the *λ*_int_ exclusively. The *λ*_e_ and *λ*_h_ can be calculated with the following equations [37]:

(1) $$\lambda_{e}=(E_{0}^{-}-E_{-}^{-})+(E_{-}^{0}-E_{0}^{0})$$

(2) $$\lambda_{h}=(E_{+}^{-}-E_{+}^{+})+(E_{+}^{0}-E_{0}^{0})$$

where $E_{+}^{0}$ and $E_{-}^{0}$ represent the energy of the neutral species with the optimized structure of the cation and anion species, while $E_{+}^{+}$ and $E_{-}^{-}$ are the energies of the cation and anion species with the optimized cation and anion structure, respectively. $E_{0}^{+}$, $E_{0}^{-}$, and $E_{0}^{0}$ are the energies of the cation, anion, and neutral species with the optimized neutral structure, respectively. Our results have thus been compared with results reported in the literature [36,37]. The *λ* for electron (*λ*_e_) and hole (*λ*_h_) were predicted at the B3LYP/6-31G(d,p) level.

## 4. Conclusions

In the study, a series of star-shaped π-conjugated organoboron compounds have been designed and systematically investigated for OLED applications. By applying density functional theory (DFT) and time-dependent DFT (TD-DFT) methodology, we calculated the FMO (HOMO and LUMO) energies, the HOMO–LUMO gaps, the absorption and fluorescence spectra, and the reorganization energies of designed molecules. It turned out that the optical, electronic, and charge transport properties are affected by the core fragments and TBGs. Our results suggest that the designed molecules can serve as luminescent materials for OLEDs. In addition, they are expected to be promising candidates for hole and/or electron transport materials.

## Figures and Tables

**Table 1:** The FMO energies *E*_HOMO_ and *E*_LUMO_, *E*_g_ (eV), and HOMO and LUMO contributions (%) of the designed molecules.

Species	HOMO				LUMO				*E*_g_
	*E*_HOMO_	CF ^a^	BB ^b^	TBG ^c^	*E*_LUMO_	CF ^a^	BB ^b^	TBG ^c^	
**1a**	−5.483	23.1	64.4	12.5	−1.693	0.4	43.8	55.7	3.789
**1b**	−6.055	23.7	68.6	7.6	−2.838	0.2	76.3	23.5	3.217
**1c**	−6.270	23.1	64.5	12.4	−2.806	0.3	33.4	66.3	3.464
**2a**	−6.244	2.3	7.1	90.7	−1.754	14.3	33.3	52.4	4.490
**2b**	−6.842	46.9	47.3	5.9	−2.794	4.3	16.7	79.0	4.049
**2c**	−7.006	45.0	43.8	11.2	−2.736	9.0	26.8	64.2	4.270
**3a**	−6.279	0.1	4.6	95.3	−2.215	34.6	35.6	29.8	4.064
**3b**	−7.440	18.7	74.1	7.2	−3.011	15.7	21.9	62.4	4.429
**3c**	−7.395	0.0	0.2	99.8	−3.066	23.1	29.8	47.1	4.328

^a^ CFs: core fragments; ^b^ BBs: benzene π-bridge fragments; ^c^ TBGs: triarylboron end groups.

**Table 2:** The longest of absorption wavelengths *λ*_abs_, the oscillator strength *f*, and assignments for the designed molecules at the TD-PBE0/6-31G(d,p)//PBE0/6-31G(d,p) level.

Species	Electronic Transitions	*λ*_abs_	*f*	Assignment
**1a**	S_0_ → S_1_	399	0.68	HOMO → LUMO (0.67) HOMO → LUMO+1 (0.17)
**1b**	S_0_ → S_1_	469	0.50	HOMO → LUMO (0.70)
**1c**	S_0_ → S_1_	433	0.72	HOMO → LUMO (0.69)
**2a**	S_0_ → S_1_	348	0.07	HOMO−2 → LUMO (0.45) HOMO−1 → LUMO (0.33)
**2b**	S_0_ → S_1_	361	0.72	HOMO → LUMO+1 (0.38) HOMO → LUMO+2 (0.45)
**2c**	S_0_ → S_1_	344	1.14	HOMO → LUMO (0.38) HOMO → LUMO+1 (0.15)
**3a**	S_0_ → S_2_	374	0.07	HOMO−2 → LUMO (0.38) HOMO → LUMO+1 (0.30)
**3b**	S_0_ → S_1_	335	0.88	HOMO → LUMO (0.39) HOMO−1 → LUMO+1 (0.36)
**3c**	S_0_ → S_3_	343	0.02	HOMO → LUMO+1 (0.48) HOMO−1 → LUMO+1 (0.23)

**Table 3:** The strongest fluorescence wavelengths *λ*_fl_, the oscillator strength *f*, and main assignments (coefficient) of the designed molecules at the TD-PBE0/6-31G(d,p)//TD-PBE0/6-31(d,p) level.

Species	Electronic Transitions	*λ*_fl_	*f*	Assignment
**1a**	S_1_ → S_0_	456	0.25	LUMO → HOMO (0.70)
**1b**	S_2_ → S_0_	496	0.49	LUMO+1 → HOMO (0.70)
**1c**	S_2_ → S_0_	499	0.57	LUMO+1 → HOMO (0.70)
**2a**	S_2_ → S_0_	364	0.41	LUMO → HOMO−1 (0.70) LUMO → HOMO−3 (0.20)
**2b**	S_1_ → S_0_	613	0.03	LUMO → HOMO (0.69) LUMO → HOMO−2 (0.10)
**2c**	S_2_ → S_0_	389	0.13	LUMO+1 → HOMO (0.70)
**3a**	S_1_ → S_0_	486	0.02	LUMO → HOMO (0.69) LUMO+2 → HOMO (0.13)
**3b**	S_1_ → S_0_	528	0.03	LUMO → HOMO (0.70)
**3c**	S_3_ → S_0_	382	0.04	LUMO → HOMO−5 (0.68) LUMO+2 → HOMO−5 (0.14)

**Table 4:** The predicted *λ*_e_ and *λ*_h_ (both in eV) of the designed molecules at the B3LYP/6-31G(d,p) level.

Species	*λ*_h_	*λ*_e_
**1a**	0.200	0.263
**1b**	0.365	0.176
**1c**	0.024	0.143
**2a**	0.069	0.156
**2b**	0.369	0.122
**2c**	0.096	0.082
**3a**	0.073	0.273
**3b**	0.270	0.134
**3c**	0.109	0.190

## Supplementary Materials

Supplementary materials can be found at www.mdpi.com/1422-0067/18/10/2178/s1.

## Abbreviations

DFT	density function theory
TD-DFT	time-dependent density function theory
OLEDs	organic light-emitting diodes
FMOs	frontier molecular orbital energies
HOMO	highest occupied molecular orbital
LUMO	lowest unoccupied molecular orbital
BBs	benzene π-bridge fragments
CFs	core fragments
TBGs	triarylboron end groups
(Mes)_2_B	(mesitylene)_2_B
(FMes)_2_B	1,3,5-tris(trifluoromethyl)benzene)_2_B
(PFB)_2_B	1,2,3,4,5-pentafluorobenzene)_2_B
DBPB	*N*-(4-(dimesitylboryl)phenyl)-*N*-phenylbenzenamine
