# Supplementary material for: Theoretical Investigations of the Photophysical Properties of Star-Shaped π-Conjugated Molecules with Triarylboron Unit for Organic Light-Emitting Diodes Applications
Source: Int J Mol Sci. 2017 Oct 18;18(10):2178. doi: 10.3390/ijms18102178 (PMC5666859; doi:10.3390/ijms18102178)
Supplement: Supplementary file 1 [file ijms-18-02178-s001.doc]

**Theoretical** **Investigations** **of** **the** **Photophysical** **Properties** **of** **Star-Shaped** **π-Conjugated** **Molecules** **with** **Triarylboron** **Unit** **for** **Organic** **Light-Emitting** **Diodes** **Applications**

**Ruifa** **Jin** **^1^**,**^2^**,*****, **Xiaofei** **Zhang** **^1^**,**^2^**, **Wenmin** **Xiao** **^1^**,**^2^** **and** **Dongmei** **Luo** **^1^**,**^2^**

^1^ College of Chemistry and Chemical Engineering, Chifeng University, Chifeng 024000, China; 15849630086@126.com(X.Z.); [xiaowenmin6868@163.com](mailto:xiaowenmin6868@163.com) (W.X.); [luodongmei1976@163.com](mailto:luodongmei1976@163.com) (D.L.)

^2^ Inner Mongolia Key Laboratory of Photoelectric Functional Materials, Chifeng University, Chifeng 024000, China

***** Correspondence: [Ruifajin@163.com](mailto:Ruifajin@163.com); Tel.: +86-0476-8300370; Fax: +86-0476-8300370

**Table S1. Calculated the longest absorption *λ*_max_ and fluorescence λ_fl_ wavelengths of N-(4-(dimesitylboryl)phenyl)-N-phenylbenzenamine by various methods with 6-31G(d,p) basis set, along with available experimental data.**

| Methods | λ_abs_ | λ_fl_ |
| --- | --- | --- |
| TD-B3LYP/6-31G(d,p) | 384 | 528 |
| TD-PBE0/6-31G(d,p) | 375 | 496 |
| TD-CAM-B3LYP/6-31G(d,p) | 323 | 360 |
| TD-wB97XD/6-31G(d,p) | 320 | 351 |
| TD-M062X /6-31G(d,p) | 336 | 375 |
| Exp^a^ | 375 | 475 |

^a^ Experimental data for N-(4-(dimesitylboryl)phenyl)-N-phenylbenzenamine in CH_3_CN were taken from ref. [24]


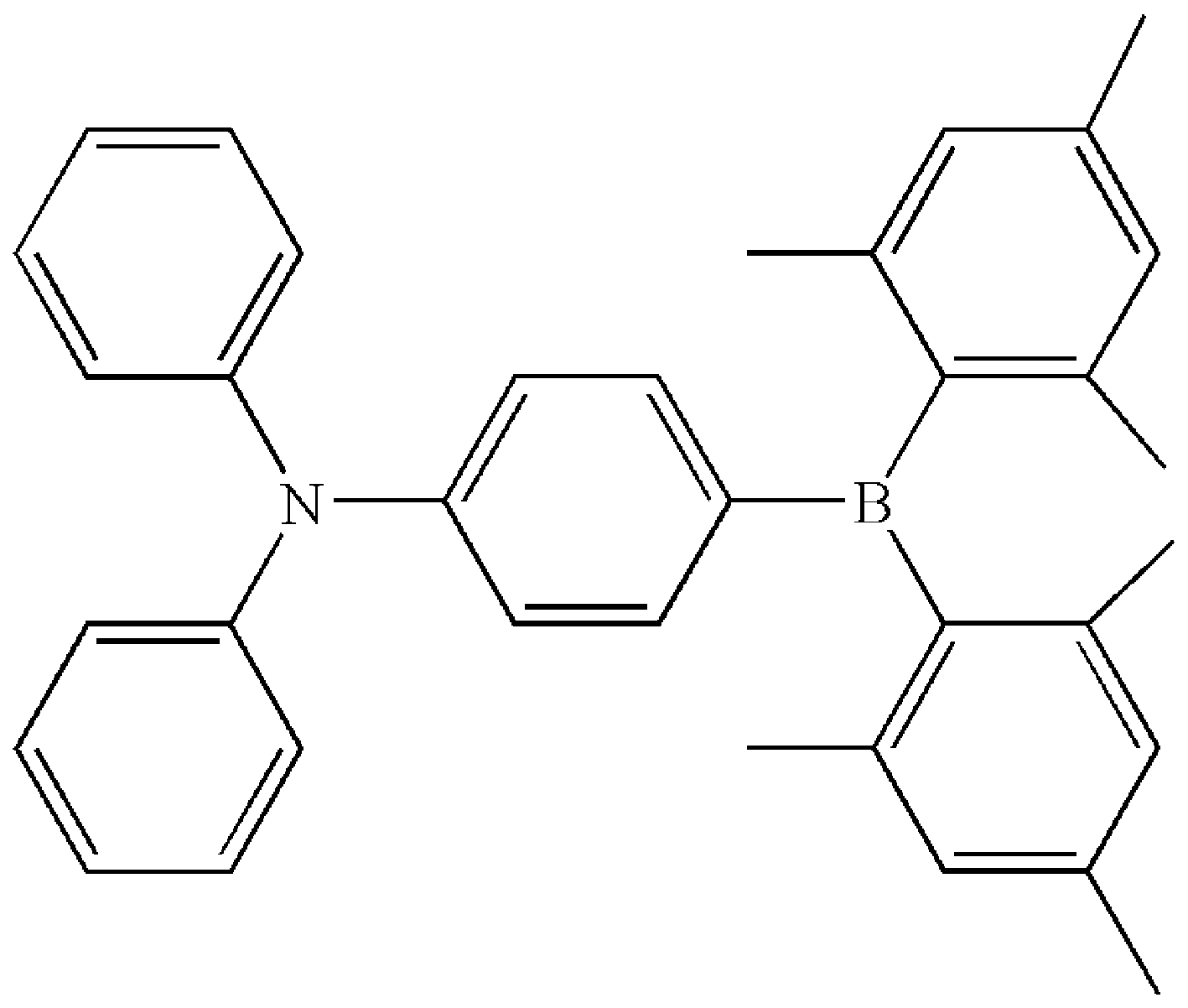


**Figure S1.** The molecular structure of N-(4-(dimesitylboryl)phenyl)-N-phenylbenzenamine (**DBPB**).
